# Complete Genome Sequence of the First KPC-Type Carbapenemase-Positive *Proteus mirabilis* Strain from a Bloodstream Infection

**DOI:** 10.1128/genomeA.00607-16

**Published:** 2016-06-23

**Authors:** Vincenzo Di Pilato, Adriana Chiarelli, Christine J. Boinett, Eleonora Riccobono, Simon R. Harris, Marco Maria D’Andrea, Nicholas R. Thomson, Gian Maria Rossolini, Tommaso Giani

**Affiliations:** a Department of Surgery and Translational Medicine, University of Florence, Florence, Italy; b Department of Medical Biotechnologies, University of Siena, Siena, Italy; c Pathogen Genomics, The Wellcome Trust Sanger Institute, Wellcome Trust Genome Campus, Hinxton, Cambridge, United Kingdom; d Department of Experimental and Clinical Medicine, University of Florence, Florence, Italy; e Clinical Microbiology and Virology Unit, Florence Careggi University Hospital, Florence, Italy; f Don Carlo Gnocchi Foundation, Florence, Italy

## Abstract

Sequencing of the *bla*_KPC_-positive strain *Proteus mirabilis* AOUC-001 was performed using both the MiSeq and PacBio RS II platforms and yielded a single molecule of 4,272,433 bp, representing the complete chromosome. Genome analysis showed the presence of several acquired resistance determinants, including two copies of *bla*_KPC-2_ carried on a fragment of a KPC-producing plasmid previously described in *Klebsiella pneumoniae*.

## GENOME ANNOUNCEMENT

*Proteus mirabilis* is one of the leading agents of urinary tract infections and can cause a number of different health care-associated infections, including mainly respiratory tract and skin infections, but also bacteremia (1). Due to a decreased susceptibility to imipenem and an intrinsic resistance to polymyxins and tetracyclines (including tigecycline), the acquisition of β-lactam resistance traits by *P. mirabilis* might seriously limit treatment options. KPC-type carbapenemases have become the most prevalent acquired carbapenemases in *Klebsiella pneumoniae* in several areas worldwide (2) and have been sporadically detected also in other *Enterobacteriaceae* and in Gram-negative nonfermenters (3–6). The acquisition of *bla*_KPC_ by *P. mirabilis* represents a rare event, and only a few descriptions have been reported to date (7–9). In this report, we announce the first complete genome sequence of a *P. mirabilis* strain carrying the *bla*_KPC-2_ gene.

*P. mirabilis* AOUC-001 was isolated in 2013 from the blood culture from an inpatient admitted to the Santa Maria Annunziata Hospital (Florence, Italy). A molecular test performed directly on positive blood culture, using the FilmArray platform (Biofire Diagnostics, UT), detected the presence of the *bla*_KPC_ gene.

Bacterial DNA was subjected to whole-genome sequencing using the MiSeq (Illumina, Inc., CA) and the PacBio RS II (Pacific Biosciences, CA, USA) platforms, which generated 4,643,660 and 1,841,288 reads, respectively**.** Short and long raw reads were processed with the PBcR hybrid assembly pipeline (10), producing 8 scaffolds (largest scaffold, 1.96 Mb; *N*_50_, 1.52 Mb; *L*_50_, 2; average G+C content, 39.42%). Genome finishing was achieved by manual inspection of the draft assembly, exploiting PacBio long reads for resolution of large repeats, and using short reads to close small gaps (11). The final *de novo* assembly generated a single molecule of 4,272,433 bp, with a raw coverage of 260×, representing the complete chromosome of AOUC-001.

A total of 3,853 coding sequences, 83 tRNAs, 22 rRNAs, and 1 type-IE clustered regularly interspaced short palindromic repeat (CRISPR)-associated (Cas) system were identified by the NCBI Prokaryotic Genome Annotation Pipeline (http://www.ncbi.nlm.nih.gov/genome/annotation_prok).

Screening for known virulence determinants (12) revealed the presence of genes associated with adhesion (mannose-resistant/*Proteus*-like fimbria [MR/P], *P. mirabilis* fimbria [PMF], and ambient-temperature fimbria [ATF] operons), motility (*flgE*, *fliFL*, *cheW*, and *flaD*), iron chelation (*hmurR2*, *znuC*, and *nrp* operon), and production of toxic compounds (*pta*, *hpmA*, and *zapA*).

The antimicrobial resistome of AOUC-001, investigated using ResFinder (13), included acquired determinants conferring resistance to aminoglycosides (*aadA1*, *aacA4*, *armA*, and *aph(3′)-Ic*), chloramphenicol (*cat* and *catA1*), trimethoprim (*dfrA1*), and sulfonamides (*sul1*). Additionally, four different β-lactamase genes (*bla*_CMY-16_, *bla*_TEM-1,_ *bla*_OXA-9_, and *bla*_KPC-2_) were detected. Interestingly, the *bla*_KPC-2_ gene was present in two copies.

Further analysis revealed that the two *bla*_KPC-2_ copies were embedded in two distinct Tn*4401a* transposons (14), arranged in a tail-to-tail configuration within the chromosome of AOUC-001. The flanking sequences of these transposons resembled the overall architecture of pKPN101-IT (accession no. JX283456), a KPC-encoding plasmid taken from a *K. pneumoniae* clinical isolate described in Italy in 2012 (15). Overall, this arrangement suggested a possible integration of a pKPN101-IT-like plasmid within the chromosome of AOUC-001, an event that might have been favored by the endemic presence of KPC-producing *K. pneumoniae* strains sharing the same setting (16).

### Nucleotide sequence accession number.

The complete genome of *P. mirabilis* AOUC-001 has been deposited at NCBI under the GenBank accession no. CP015347.
